# Genome-wide phenotypic RNAi screen in the *Drosophila* wing: global parameters

**DOI:** 10.1093/g3journal/jkab351

**Published:** 2021-10-02

**Authors:** Ana López-Varea, Cristina M Ostalé, Patricia Vega-Cuesta, Ana Ruiz-Gómez, María F Organista, Mercedes Martín, Covadonga F Hevia, Cristina Molnar, Jesús de Celis, Joaquim Culi, Nuria Esteban, Jose F de Celis

**Affiliations:** Centro de Biología Molecular “Severo Ochoa,” CSIC and Universidad Autónoma de Madrid, Madrid 28049, Spain

**Keywords:** phenotype, wing, screen, RNAi

## Abstract

We have screened a collection of *UAS-RNAi* lines targeting 10,920 *Drosophila* protein-coding genes for phenotypes in the adult wing. We identified 3653 genes (33%) whose knockdown causes either larval/pupal lethality or a mutant phenotype affecting the formation of a normal wing. The most frequent phenotypes consist of changes in wing size, vein differentiation, and patterning, defects in the wing margin and in the apposition of the dorsal and ventral wing surfaces. We also defined 16 functional categories encompassing the most relevant aspect of each protein function and assigned each *Drosophila* gene to one of these functional groups. This allowed us to identify which mutant phenotypes are enriched within each functional group. Finally, we used previously published gene expression datasets to determine which genes are or are not expressed in the wing disc. Integrating expression, phenotypic and molecular information offers considerable precision to identify the relevant genes affecting wing formation and the biological processes regulated by them.

## Introduction

The availability of complete genome annotations in model organisms together with the development of knockdown techniques and mutant analysis opens the possibility of genome-wide phenotypic descriptions (St Johnston 2002; Dietzl *et al.* 2007). One goal of such analyses is to provide a new dimension, the mutant phenotype, to the molecular annotation of genomes. The phenotype of individual mutations or knockdowns informs about the requirements of a gene in a particular process and serves as an entry point to further in-deep characterization of its functional roles. In general, most genes are expressed and participate in many developmental stages and tissues, and consequently, each mutant phenotype includes a variety of components related to the specific characteristics of the tissue under scrutiny. Reconstructing the steps linking a mutation or knockdown to a morphological phenotype in a particular tissue is helped by our previous understanding of the processes involved in the development of that tissue. In turn, the analysis of morphological phenotypes allows the identification and further characterization of these developmental operations.

We have screened *UAS-RNAi* lines targeting 10,920 *Drosophila* genes in the fly wing, a tissue for which there is a wealth of information regarding the main steps and components of its development (de Celis 2003; Beira and Paro 2016). Imaginal discs are epithelial tissues that give rise during metamorphosis to the adult structures of the fly. The wing imaginal disc differentiates half of the thorax and one wing. Its development initiates during embryogenesis with the specification of a primordium composed of approximately 40 cells (Ostalé *et al.* 2018). Subsequently, these cells invaginate and start proliferating, forming at the end of the third larval instar a structure composed of approximately 50,000 cells. At this stage, each cell in the disc is genetically programmed to differentiate during pupal development specific adult structures, either the cuticle that forms the wing and thorax or the different elements that decorate the body and appendage, including sensory organs and wing veins. The development of the wing disc involves considerable cell proliferation, and this is accompanied by a progressive regionalization of the disc into the different presumptive regions of each adult structure (Ostalé *et al.* 2018). This process is based on a variety of gene regulatory networks leading to the generation of restricted spatial patterns of gene expression. Common components of these gene regulatory networks are transcription factors and a set of signaling pathways. The integration of these two elements underlies the generation and expansion of gene expression domains during the development of the wing disc.

Cell proliferation and differentiation, as well as pattern formation, are common developmental processes in multicellular tissues controlled by evolutionary conserved batteries of genes. We expect that genes involved in the regulation of these processes would affect wing formation by altering its size or the spatial distribution of differentiated elements. This is indeed the case for genes affecting the cell cycle, which insufficiency results in the formation of smaller wings (Edgar 2006; Cruz *et al.* 2009). It is also the case for genes encoding components of several signaling pathways, whose mutations result in alterations in the pattern and/or differentiation of veins, sensory organs, or the wing margin (Molnar *et al.* 2011). In addition, we expect that knockdown of genes participating in general cellular functions such as transcription, translation, protein trafficking, or metabolism might also cause morphological alterations in the wing, for example as a result of compromised cell viability. Finally, the *Drosophila* genome contains a considerable fraction of genes that are not present in other organisms (Adams *et al.* 2000), and in these cases, mutant phenotypes are fundamental data to initiate their functional characterization.

Many studies have shown that the wing disc is an extremely reactive structure to genetic and developmental perturbations (Repiso *et al.* 2011; Beira and Paro 2016). In addition, the adult wing is morphologically simple, in essence, the result of the combination of two layers of cuticle with a precise size and shape, but contains enough information in terms of patterned elements (veins and sensory organs) to identify even subtle alterations to its size and morphology as a result of changes in gene expression (de Celis 2003). In this work, we combine the results of a global RNAi screen with gene expression data and with a simplified molecular gene annotation with the aim of generating a searchable dataset conveying the main phenotypic consequences of the knockdown of a substantial fraction of the *Drosophila* genome.

## Materials and methods

### *Drosophila* strains

We made Gal4/UAS-RNAi combinations using the Gal4 drivers *sal^EPv^-Gal4* (Cruz *et al.* 2009), *nub-Gal4*, and *sd-Gal4* (Calleja *et al.* 1996). The UAS-RNAi lines were from the Vienna *Drosophila* Resource Center (VDRC), National Institute of Genetics Fly Stock Center (NIG-Fly), and Bloomington *Drosophila* Stock Center (BDSC; see Supplementary Table S1). Flies were raised at 25°C (unless otherwise stated) in fly medium containing Glucose (50 gr/L), Agar (7.86 gr/L), wheat flour (35.7 gr/L), yeast (71.4 gr/L), Methylparaben (2.8 mL/L), and Propionic acid (4.3 mL/L). Adult flies of *Gal4/UAS-RNAi* genotype were scored under the dissection microscope, and selected wings of *UAS-Dicer2/+; nub-Gal4/UAS-RNAi and UAS-Dicer2/+; sal^EPv^-Gal4/UAS-RNAi* combinations (approximately 800) were mounted in Lactic acid-Ethanol (6:5) for microscopic examination. Pictures were taken using a Spot digital camera coupled to a Zeiss Axioplan microscope (5X objective). Pictures were captured and the background set to white using Photoshop v21.2 (Adobe™).

### Gene expression analyses

#### RNA-Seq

We took advantage of published RNA-Seq data obtained from dissected wing imaginal discs (Flegel *et al.* 2016). In particular, reads from run SRR3478156, corresponding to control larvae expressing Gal4/GFP, were quantified using Sailfish 0.7.6.0 running at the Galaxy platform. *Drosophila melanogaster* dm6 transcriptome was used as reference. Estimated relative expression levels were expressed as transcripts per million (TPM).

#### Affymetrix microarrays

Wing imaginal discs (40 discs per sample in three replicates) were dissected and stored at −80°C. Total RNA was extracted using the guanidinium isothiocyanate method (TRIzol reagent; Invitrogen, Carlsbad, CA, USA), followed by purification using an RNeasy column (Qiagen, Valencia, CA, USA). Each RNA preparation was tested for degradation using the Agilent 2100 Bioanalyzer (Agilent Technologies, Palo Alto, CA, USA). cDNA was synthesized from total RNA using One-Cycle target labeling and control reagents (Affymetrix, Santa Clara, CA, USA) to produce biotin-labeled cDNA. The cDNA preparations (10 μg) were fragmented (94°C, 35 min) into 35–200 bases in length and hybridized to the GeneChip™ *Drosophila* Genome 2.0 Array (Affymetrix) which contains 18,880 probe sets, analyzing over 18,500 transcripts. Each sample was added to hybridization solution containing 100 mM 2-(N-morpholino) ethanesulfonic acid, 1 M Na+ and 20 mM EDTA, with 0.01% Tween-20 to a final cDNA concentration of 0.05 μg/mL. Hybridization was performed for 16 h at 45°C. Each microarray was washed and stained with streptavidin-phycoerythrin in a Fluidics station 450 (Affymetrix) and scanned at 1.56 μm resolution in a GeneChip Scanner 3000 7G System (Affymetrix). Images were acquired and analyzed using GeneChip Operating Software (GCOS). Microarray processing, hybridization, and initial statistical analysis were performed by the Genomics unit at the Centro Nacional de Biotecnología. Deeper data analysis was performed at the Centro de Biologia Molecular “Severo Ochoa.” We used the average expression data for third instar control wing discs (*sd-Gal4/UAS-GFP*) described in Organista *et al.* (2015).

#### In situ hybridization

We used a collection of 635 pictures of *in situ* hybridization experiments carried out in our laboratory and published in Molnar *et al.* (2006, 2012), Organista *et al.* (2015), and Hevia *et al.* (2017). The expression patterns were classified as no expression (NE), generalized expression (GEN), and patterned expression (PAT). For a set of 562 genes, we compared the expression levels (RNA-Seq and Microarray) with the expression observed by *in situ* hybridization. We defined as “1” when at least one quantitative data were concordant with the *in situ* and “0” when there was no concordance between the three experiments (Supplementary Table S2).

### Gene ontology and InterPro analysis

We compiled all Gene ontology (GO) annotations and InterPro (IP) domains for all *Drosophila* coding genes using Flymine (Lyne *et al.* 2007) and Flybase (Thurmond *et al.* 2019). All available descriptions were summarized in a single term indicating one functional class. These classes were “Cell adhesion” (CA), “Cell death” (CD), “Cuticular differentiation” (CUT), “Cytoskeleton organization” (CYT), “Cell division” (DIV), “Ribosome function” (RIB), “Cell signaling” (SIG), “Transport across cell membranes” (TRA), “Protein trafficking” (PTR), “Cellular metabolism” (MET), “Immune Responses” (IMM), “DNA Biology” (DNA), “RNA Biology” (RNA), and “Protein modifications” (PRO). A list of all abbreviations used in this manuscript is presented in Table 1. The primary annotation was further curated using the “Gene group” classification available from Flybase and individual gene descriptions also available in Flybase (Thurmond *et al.* 2019). Genes without any information based on sequence were classified as “CG,” and genes for which there is at least one IP domain as CGh.

**Table 1:** List of abbreviations used to define wing phenotypes and molecular classes

	Phenotypic description		Phenotypic description		Molecular classes
nec	Necrotic wing disc	PL	Pupal lethal	CG	Coding gene with no homology
nW	Wing missing	EPL	Early Pupal Lethal	CG(h)	Coding gene with IP domain
S	Smaller wing size	LL	Larval lethal	CA	Cell adhesion
S(L)	Larger wing size	V+	Ectopic wing veins	CD	Cell Death
S-P	Size and pattern defects	V+(N)	Thicker wing veins	CUT	Cuticle
wt	Normal wing	V−	Loss of wing veins	CYT	Cytoskeleton
WS	Wing shape defects	(L2/L3/L4/L5)	Longitudinal veins 2, 3, 4, and 5	DIV	Cell division
WS(ds)	Wing shape defects: broader	cv	Crosveins	DNA	DNA biology
WS (lc)	Wing shape defects: lanceolate	acv	Anterior crosvein	IMM	Immunology
WS (dp)	Wing shape defects: shorther	pcv	Posterior cross vein	MET	Metabolism
WS (hinge)	Wing hinge defects	WD	Wing differentiation defects	PRO	Protein Biology
WS (Cy)	Wing shape defects: Curled wings	CD	Trichome differentiation defects	PTR	Protein transport
WS (haltere)	Wing to halter transformation	ds	Broader wing; escapers of WF(s)	RIB	Ribosome
WM	Integrity of the wing margin	Q+	Ectopic bristles	RNA	RNA biology
WF	Wings folded	Q+	Loss of wing margin bristles	SIG	Cell signaling
WA	Wing surface adhesion	WP	Wing cuticle pigmentation defects	TRA	Transport across membranes
(s)	Strong phenotype	(w)	Weak phenotype		

## Results and discussion

### Global data of the RNAi screen

The *Drosophila* genome includes 13,957 RNA protein-coding genes and a total of 3867 RNA nonprotein-coding regions, including lncRNA, asRNA, CR, tRNA snoRNA, mir-RNA, and rRNA (Thurmond *et al.* 2019; Supplementary Table S1). We obtained *UAS-RNAi* strains, mostly from VDCR, but also from NIG-Fly and BDSC (Supplementary Table S1), targeting 10,920 protein-coding mRNAs. The design of the VDCR RNAi library was based on Release 4.3 of the *Drosophila* genome, and since then a large number of genes have been added to the current annotation (R6.37). We noticed that the set of genes we did not include in our analysis (3037 genes) is enriched in proteins of unknown function (CG and CGh; 1284 genes, 42% *vs* 23% in the set of 2475 CG and CGh genes that we analyzed). Most of these genes comprise small open reading frames recently added to the genome annotation (Couso and Patraquim 2017). Furthermore, we estimated that 29% of the genes we did not analyze are not expressed in the wing disc (1572 out of 5433 genes), compared with 16% of genes not analyzed that were counted as expressed in the wing disc (1379/8415 genes; see below).

**Figure 1:**
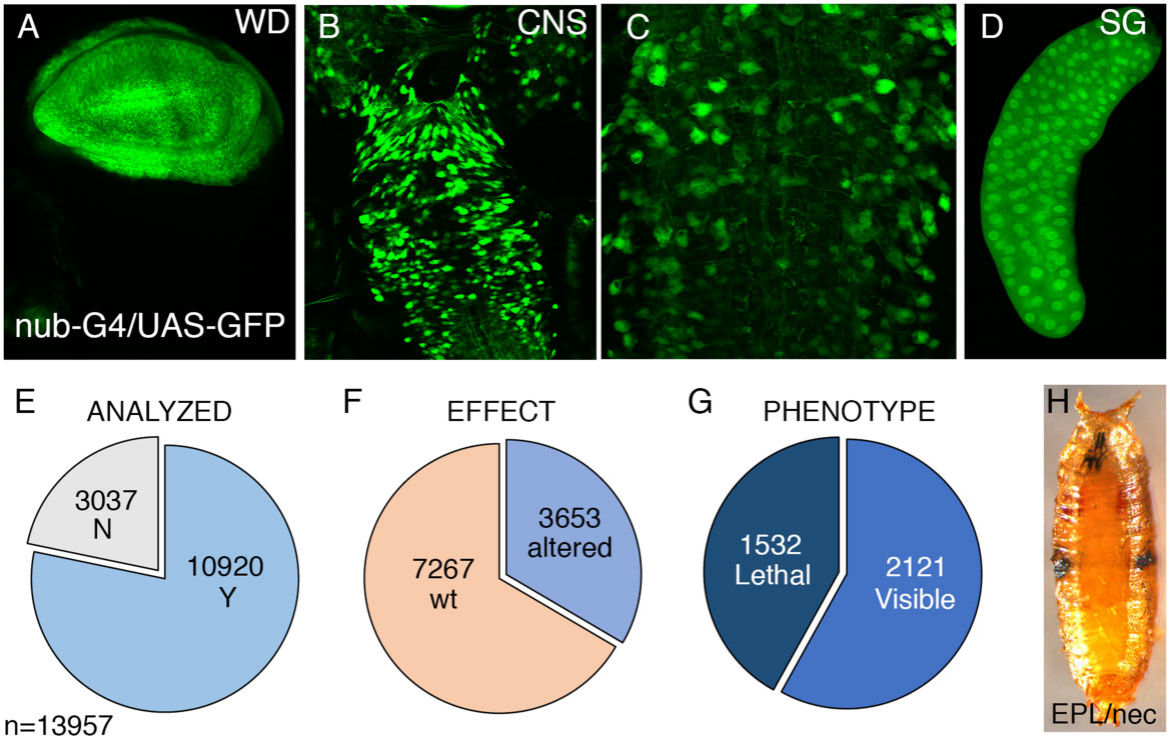
Overall results of the screen. (A–D) Expression of the *nub-Gal4* driver (GFP; green) in the wing imaginal disc (A), central nervous system (B, C), and salivary gland (D) in third instar larvae of *nub-Gal4/UAS-GFP* genotype. (C) A higher magnification of B. (E) Number of genes analyzed (blue) and not analyzed (gray) in *UAS-Dicer2/+; nub-Gal4/UAS-RNAi* combinations. (F) Number of genes with (blue) and without (red) a lethal or visible phenotype in *UAS-Dicer2/+; nub-Gal4/UAS-RNAi* flies. (G) Number of lethal (dark blue) and viable lines with a visible phenotype (light blue) in *UAS-Dicer2/+; nub-Gal4/UAS-RNAi* combinations. (H) Early pupal lethal with necrotic wings phenotype (*UAS-Dicer2/+; nub-Gal4/UAS-CG4294-i*) (I).

We crossed *UAS-RNAi* males from these 10,920 strains with *UAS-Dicer2; nub-Gal4* virgin females. All these crosses were made in a *Dicer2* overexpression background to increase the efficiency of RNA interference. The *nub-Gal4* driver is expressed in the wing blade and hinge region of the wing imaginal disc and is also expressed in many cells of the larval central nervous system and salivary glands (Figure 1, A–D). The expression of *nub-Gal4* during pupal development is progressively lost from the interveins, becoming restricted to the future veins at approximately 12 h after puparium formation (data not shown). The complete results of the screen are presented in Supplementary Table S1. This table also contains the molecular annotation for each gene (see below), its expression level in the wing disc (see below), and the particular *UAS-RNAi* strain used in each case. Out of 10,920 genes analyzed (Figure 1E), we obtained for 3653 genes either lethality or a mutant phenotype in the wing (Figure 1, F and G), indicating that expression of RNAi reveals functional requirements for an estimated 33% of the *Drosophila* protein-coding genes (Table 2). Lethality (1532 combinations; 14%; Table 2) was manifested in late third instar larvae and through pupal development. Larval lethality includes many cases where there is an extended third instar larval period with a total (132 of 1532) or partial (269/1532) failure to progress to the pupal stage. The majority of lethal combinations (621/1532) displayed early pupal lethality accompanied by necrotic masses of tissue in the position of the developing wing discs (“EPL/nec”; Figure 1H).

**Table 2:** Number of genes in each molecular class (N°), analyzed genes (Done), genes with (YES) or without (NO) a phenotype and number of each phenotype identified in the screen

	PRO	MET	DNA	TRA	SIG	RNA	PTR	CYT	CUT	CA	RIB	IMM	DIV	CD	CGh	CG	Genome
N°	1,689	1,631	1,598	954	880	851	659	513	387	277	235	216	245	63	1,675	2,084	13,957
Done	1,364	1,338	1,253	797	755	702	609	431	312	250	197	169	218	50	1,294	1,181	10,920
Yes	392	385	510	199	271	391	240	165	88	87	178	41	114	17	299	276	3,653
No	972	953	743	598	484	311	369	266	224	163	19	128	104	33	995	905	7,267
LL/EPL/PL	161	174	193	87	72	247	108	54	37	19	150	15	40	7	87	81	1,532
nW	36	21	78	13	22	38	44	16	3	5	9	3	21	0	17	9	335
S-P	21	22	38	11	30	22	54	16	7	5	6	2	9	1	9	8	261
S	105	90	141	36	98	85	43	61	20	16	17	5	46	4	82	52	901
V+	57	32	56	23	40	38	33	26	7	8	8	4	5	1	43	39	420
V−	21	4	35	6	37	8	10	9	2	7	2	1	2	0	9	18	171
WA	66	53	57	38	38	34	38	30	10	29	3	13	14	3	74	71	571
WM	11	12	24	9	22	15	8	7	2	2	1	4	7	0	19	13	156
WD	43	45	37	19	14	26	29	10	12	15	24	3	6	1	44	30	358
WS	6	5	14	3	5	1	0	1	4	3	0	1	2	1	12	5	63
WP	3	6	2	3	0	1	1	1	1	1	0	1	0	0	1	0	21
Q	1	3	3	0	3	0	1	1	0	0	0	0	0	0	1	2	15
CD	4	3	20	0	2	3	2	9	0	2	0	1	26	0	1	1	74
Expression (Y)	1,018	1,089	1,219	477	493	818	552	375	119	184	224	106	225	54	888	629	8,470
Expression (N)	685	536	394	477	383	31	105	135	268	93	11	110	19	9	789	1,390	5,435
Ex N Phe Y	96	94	75	65	62	8	18	22	49	17	5	19	6	3	102	159	800
%Exp	60.3	66.8	76.283	50.0	56.0	96.1	83.8	73.1	30.7	66.4	95.3	49.1	91.8	85.7	53.0	31.2	61.0

Number of genes expressed (Expression Y) and not expressed (Expression N) in the wing disc, number of not expressed genes with a knockdown phenotype (Exp N Phe Y) and percentage of genes expressed in each molecular class (%Exp).

### Visible wing phenotypes

**Figure 2:**
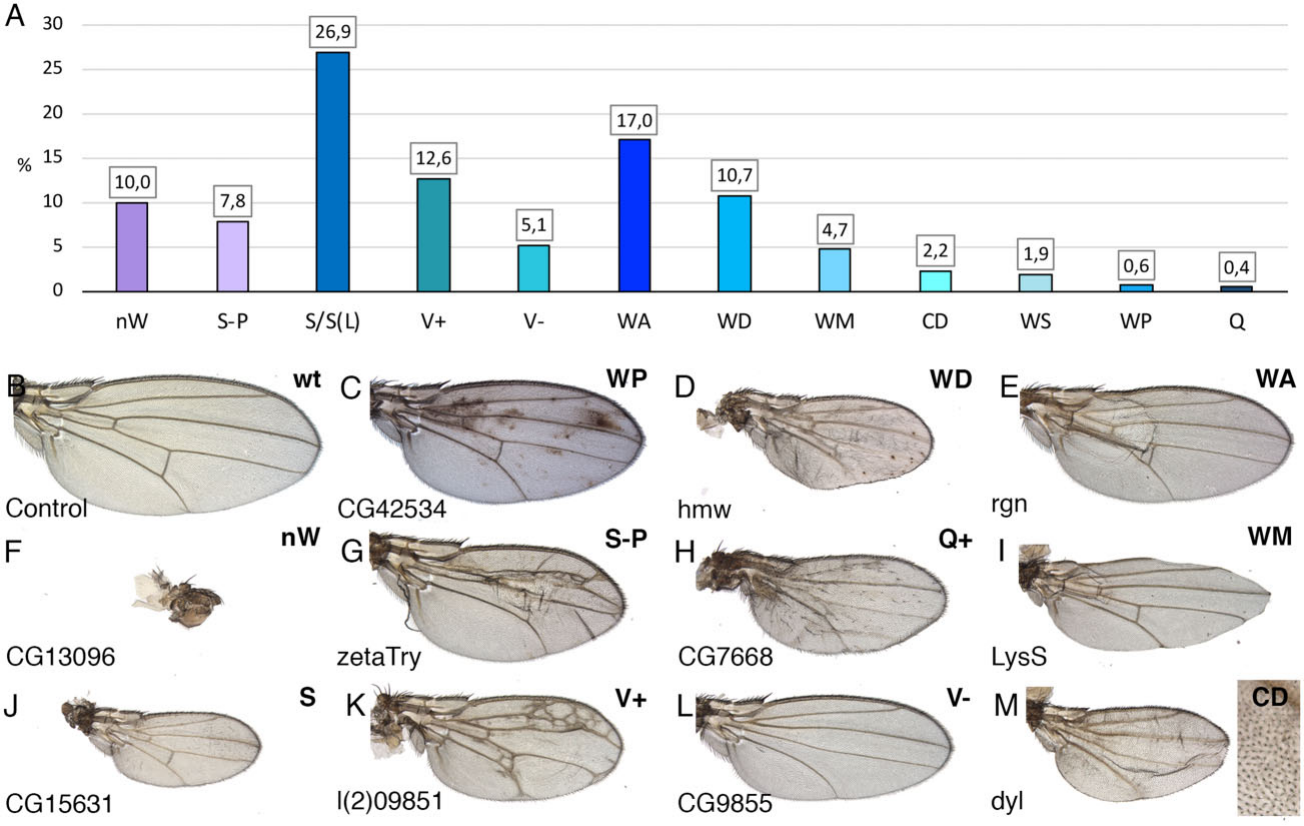
Frequency of different wing phenotypes. (A) Overall frequency of adult phenotypes distributed in the groups “nW” (failure to form the wing), “S-P” (changes in the size of the wing and relative positions of the veins), “S/S(L)” (wing size alterations), “V+” (ectopic or thicker veins), “V−” (loss of veins), “WA” (failures in the adhesion between the dorsal and ventral wing surfaces), “WD” (altered wing cuticular differentiation), “WM” (defects in the wing margin), “CD” (changes in cell size or trichome differentiation), “WS” (shape of the wing), “WP” (changes in wing pigmentation), and “Q” (differentiation of ectopic bristles in the wing surface, “Q+”, or loss of bristles in the wing margin, “Q−”). (B–M) Representative examples of wings illustrating the main observed phenotypes. Wild-type control wing (*UAS-Dicer2/+; nub-Gal4/UAS-GFP*; B), *UAS-Dicer2/+; nub-Gal4/UAS- CG42534-i* (“WP”; C), *UAS-Dicer2/+; nub-Gal4/UAS-hmw-i* (“WD”; D), *UAS-Dicer2/+; nub-Gal4/UAS-rgn-i* (“WA”; E), *UAS-Dicer2/+; nub-Gal4/UAS-CG13096-i* (“nW”; F), *UAS-Dicer2/+; nub-Gal4/UAS-zetaTry-i* (“S-P”; G), *UAS-Dicer2/+; nub-Gal4/UAS-CG7668-i* (“Q+”; H); *UAS-Dicer2/+; nub-Gal4/UAS-LysS-i* (“WM”; I), *UAS-Dicer2/+; nub-Gal4/UAS-CG15631-i* (“S”; J), *UAS-Dicer2/+; nub-Gal4/UAS-l(2)09851-i* (“V+”; K), *UAS-Dicer2/+; nub-Gal4/UAS-CG9855-i* (“V−”; L) and *UAS-Dicer2/+; nub-Gal4/UAS- dyl-i* (“CD”; M). Inset in M is a higher magnification of a lateral region of the wing.

Wing phenotypes were observed in escapers of some of these combinations and in many viable combinations (*n* = 2121; Figure 1G;Supplementary Tables S1 and S2). We tried to summarize the main phenotypic components of each mutant wing using a simplified nomenclature (Table 1) based on the following abbreviations: “no-wing” includes all cases of adult flies or pharate pupa in which the wing is not present or rudimentary (“nW”; Figure 2, A and F). “Size” refers to wings with reduced wing size, but where the distribution of veins was normal or near normal (“S”; Figure 2, A and J). Those rare cases where the wings were larger than normal were described as “S(L).” “Size and Pattern” refers to wings where both the size and the relative distribution of veins along the anteroposterior axes are altered (“S-P”; Figure 2, A and G). These wings could also display the loss of some longitudinal veins, but always associated to a significant reduction of wing size and a general misposition of the remaining longitudinal veins. Wings in which some vein stretches are missing but without strong defects in wing size or in the position of the remaining veins were named “V−” (Figure 2, A and L). Conversely, wings differentiating excess of veins were defined “V+” (Figure 2, A and K). Most of these cases correspond to wings differentiating ectopic veins located between the longitudinal veins L2 and L3 or between the veins L4 and L5. In a minority of cases, the wings do not differentiate ectopic veins, but the veins are thicker than normal. This phenotype was named, following the characteristic vein thickening caused by *Notch* gene insufficiency “V+(N).” The overall shape of the wing surface could also be altered without changes in the pattern of veins. These phenotypes were defined as “WS” (wing shape defects) and include wings that are narrowed along the anteroposterior axes, a phenotype reminiscent of the mutant lanceolate “WS(lan),” wings shortened along the proximo-distal axes, reminiscent of the dumpy viable wing phenotype “WS(dp),” wings broader than normal, reminiscent of the *daschous* viable phenotype (ds), curved wings “WS(Cy)” and wings transformed into haltere [WS(haltere)]. Other phenotypes include defects in the integrity of the wing margin (“WM”; Figure 2, A and I), the formation of wing blisters, likely caused by defects in dorso-ventral wing surface adhesion (“WA”; Figure 2, A and E), defects in wing pigmentation (“WP”; Figure 2, A and C), changes in the number, size, spacing, or differentiation of the trichomes, the hairs formed by each wing cell (cell differentiation; “CD”; Figure 2, A and M) and defects in the number of bristles in the wing (Q− and Q+ to indicate loss and ectopic bristles in the wing margin and wing surface, respectively; Figure 2, A and H). Finally, other defects that we were unable to classify in these categories were defined as wing differentiation defects (“WD”; Figure 2, A and D). They include incomplete unfolding of the wing surfaces, the appearance of necrotic patches, wing cuticle with abnormal appearance, or lack of rigidity. All abbreviations used through the manuscript are presented in Table 1.

In general, we notice that flies of the same genotype show low variability among individuals, allowing us to define each wing using the nomenclature defined above and presented in Table 1. Some of these phenotypes can appear simultaneously in wings of the same genotype (Figure 2, C–M). For the purpose of quantification, we considered each phenotypic component appearing in the same wing as an independent event (Figure 2A). The only exception was the case of genotypes with “no-wing phenotype” (“nW”), where the presence of additional phenotypic annotations corresponds to the use of other drivers, mostly *sal^EPV^-Gal4*, and was not included in the quantification. The most frequent phenotypes were those related to alterations in the size of the wing [26.9% “S/S(L)”], differentiation of the veins (17.7% “V+” and “V−”), dorso-ventral wing surface adhesion (17% “WA”), and wing cuticle differentiation defects (10.7% “WD”). Also frequent were phenotypes of wing loss (10% “nW”) and defects in wing size and vein patterning (7.8% “S-P”). The overall frequency of phenotypes in shown in Figure 2A and some representative examples phenotypes are shown in Figure 2, C–M.

### Prevalence of knockdowns without phenotypic consequences in the wing

Our data indicate a strong occurrence of genes whose knockdowns fail to produce either lethality or a visible phenotype (66% of the genes analyzed, corresponding to 7267 genes). There are many reasons that could contribute to this high fraction of genes whose function appears dispensable for wing imaginal disc development. In the first place, the number of inactive RNAi lines are estimated to comprise between 15% and 40% in different *UAS-RNAi* collections (Dietzl *et al.* 2007; Perkins *et al.* 2015). A second reason is insufficient knockdown efficiency, which could result in false negatives. The efficiency of knockdown is specific for each individual RNAi, and in a random collection of 64 *UAS-RNAi/Act-Gal4* viable combinations, it varies almost linearly from 95% to 10% reductions in mRNA amount (Dietzl *et al.* 2007). Furthermore, only an estimated 38% of these combinations resulted in a reduction of mRNA level below 25% of normal expression (Dietzl *et al.* 2007). Complementary, it is expected that only a reduction in mRNA levels below a certain threshold would result in a phenotype, and this threshold is expected to be specific for each gene. For example, mutations in genes encoding proteins that form part of the ribosome behave as haplo-insufficient, because a 50% reduction in gene dose results in a *Minute* dominant phenotype (Marygold *et al.* 2007). In this manner, it is expected for these genes that even a weak or moderate efficiency of knockdown results in an altered phenotype. Consistently, we find that RNAi directed against genes encoding components of the ribosome have the highest rate of effects. The number of haplo-insufficient genes in *Drosophila* beyond ribosomal genes is very low and includes a handful of genes mostly encoding components of the Notch signaling pathway, indicating that for the majority of *Drosophila* genes a reduction larger than 50% is needed to obtain a mutant phenotype. A third reason explaining a fraction of the *nub-Gal4/UAS-RNAi* combinations that give no mutant phenotype is gene redundancy. The number of gene duplications present in the *Drosophila* genome is very high (Osada and Innan 2008; Bao *et al.* 2018), and, for example, several *Drosophila* gene complexes encode two or more transcriptional regulators that play similar roles and are expressed in the same spatial pattern during wing imaginal development. Some examples of these gene complexes that play significant roles during wing patterning are the *achaete-scute* (García-Bellido and de Celis 2009), *spalt* (de Celis and Barrio 2009), *Iroquois* (Cavodeassi *et al.* 2001), *Enhancer of split* (Schrons *et al.* 1992), and *knirps* gene complexes (Lunde *et al.* 1998). Finally, many genes playing important functional roles during embryonic development and all those which are tissue-specific are likely not expressed in the wing disc, and consequently, it is expected that the expression of RNAi directed against them in the wing disc result in normal adult flies. Definitive results concerning gene requirements in a tissue of interest can only be obtained by systematically evaluating gene knockouts, something that will be possible by the development of genome-wide libraries allowing CRISPR conditional gene disruption (Port *et al.* 2020). It is expected that such approaches would be more efficient than RNAi to reveal functional requirements, as RNAi only causes hypomorphic conditions that may be insufficient to cause a phenotype. Despite this and other limitations inherent to an experimental approach based in RNAi expression, the phenotypes we observed allow a glimpse into the potential function of a large collection of genes that might be later extended and validated by subsequent monographic analyses.

### Correlation between gene expression and knockdown phenotypes

**Figure 3:**
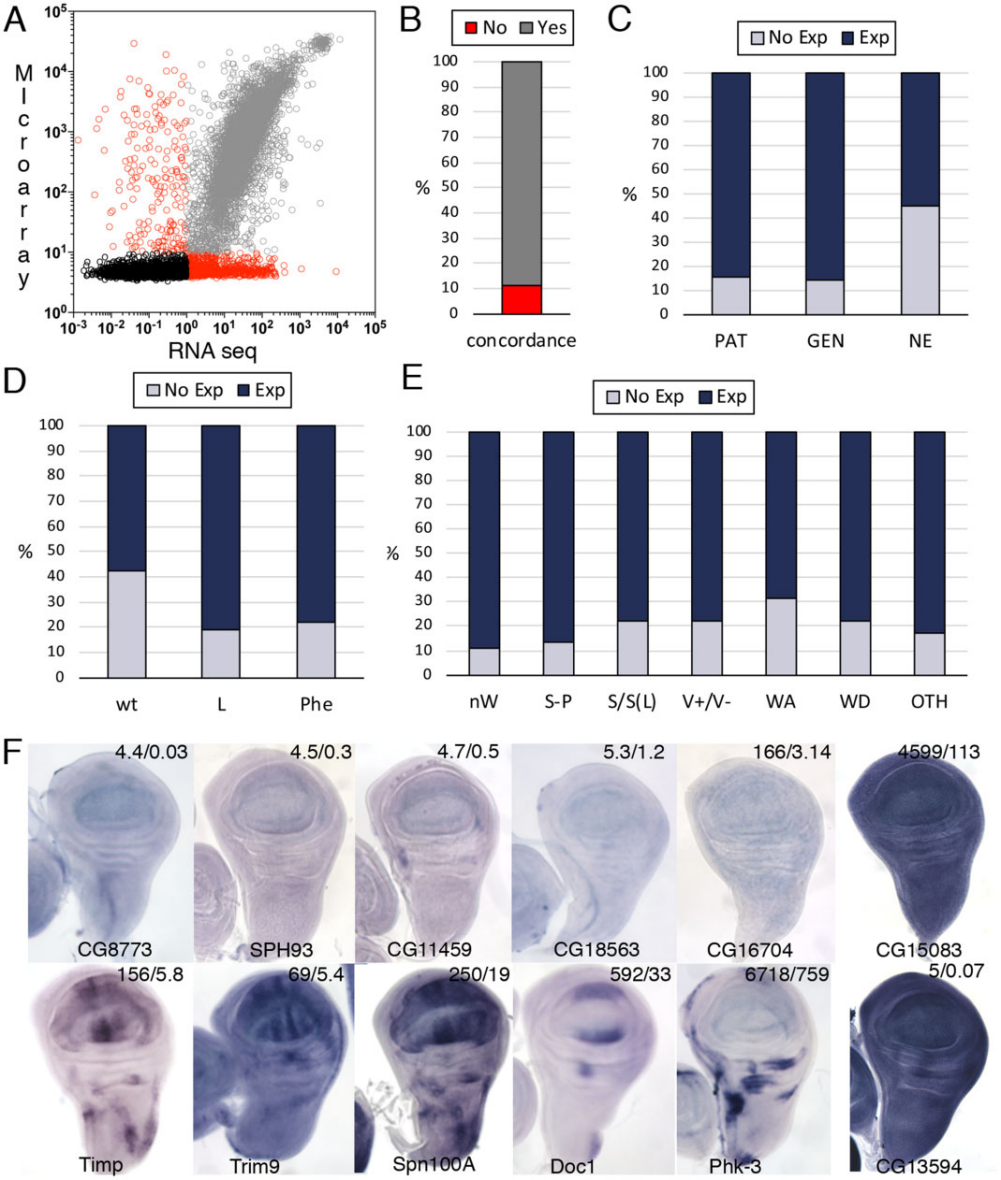
Gene expression and phenotypic correlations. (A) Logarithmic representation of the expression values obtained from Affymetrix (Microarray) and RNA-Seq expression values (values equal to 0 are not represented in the logarithmic scale). Nonconcordant data are shown as red circles (*R*^2^ = 0.0) and concordant data as black (*R*^2^ = 0.02) and gray circles (*R*^2^ = 0.69) for genes not expressed or expressed, respectively. (B) Percentage of genes showing expression or not expression in both Affymetrix and RNA-seq data (gray section of the column) and genes with nonconcordant values of expression (red column section of the column). (C) Expression detected by *in situ* hybridization were grouped in spatial PAT, GEN, and NE. For each group dark blue section of each column represents the percentage of genes defined as expressed in the wing disc and light blue section of each column those not expressed in Affymetrix or RNA-Seq experiments. (D) Percentage of genes defined as expressed (dark blue section of each column) or not expressed (light blue columns) without a knockdown phenotype (wt), lethality (L), or a visible phenotype (Phe) in *UAS-Dicer2/+; nub-Gal4/UAS-RNAi* combinations. (E) Percentage of genes defined as expressed (dark blue section of each column) or not expressed (light blue section of each column) resulting in loss of wing (“nW”), size and pattern defects (“S-P”), size defects (“S/S(L)”), loss or gain of vein phenotypes (“V+/V−”), loss of dorso-ventral adhesion (“WA”), defects in wing differentiation defects (“WD”), and other less frequent phenotypes (“OTH”). (F) Examples of *in situ* hybridization patterns showing the levels of expression detected in Affymetrix and RNA-Seq experiments (Affymetrix/RNA-Seq) in the up-right corner of each picture.

In order to correlate our results with gene expression in the wing disc, we used two global data sets obtained from Affymetrix microarrays generated by us and from RNA-Seq published by Flegel *et al.* (2016). We obtained expression data for 13,848 genes, for which there were expression values in both dataset for 79% of genes (*n* = 10,994). To evaluate the consistency of these two independent sets, we first compared the expression levels of these 10,994 genes in both datasets and found a significant linear correlation between them (*R*^2^ = 0.63 by Pearson correlation, *P* < 0.0001; Figure 3A). When we arbitrarily set a threshold cutoff for expression in the wing disc of 1 for Affymetrix (average expression value) and 10 for the RNA-Seq (TPM value) the results were concordant (expression or not expression in both datasets) for 89% of the 10,994 genes analyzed (Figure 3B). Using these expression data, and considering that a gene is transcribed when its expression value is above our arbitrary threshold cutoff in either dataset, we estimated that 61% of *Drosophila* protein-coding genes are expressed in the wing disc (Table 2; Supplementary Table S1). We also compared the expression levels detected by microarray and RNA-Seq with images of *in situ* hybridization for 562 genes that we published as supplementary information in Molnar *et al.* (2006, 2012), Cruz *et al.* (2009), Organista *et al.* (2015), and Hevia *et al.* (2017). This comparison serves as an independent corroboration of confidence when classifying genes as expressed or not expressed in the wing disc. We found that the fraction of genes classified as expressed in the wing disc for which we could detect expression by *in situ* hybridization varies from 86% (348/406) to 84% (75/89) for genes ubiquitously expressed (GEN) and expressed in a restricted pattern (PAT), respectively (Figure 3C). Only 45% (30 out of 67) of genes considered as not expressed in the wing disc are also not detected by *in situ* hybridization (Figure 3C).

We then compared expression and phenotypic class for a collection of 10,803 genes. For those genes whose knockdowns result in wild-type wings, we found that 42% (*n* = 3060) were estimated as not expressed in the wing disc (Figure 3D). This fraction is much smaller for genes with a mutant phenotype. For example, only 19% (*n* = 292) of genes whose knockdown results in lethality are not expressed in the wing disc (Figure 3D). A similar fraction of 22% (*n* = 496) combinations resulting in a visible wing phenotype corresponds to genes not expressed in the wing disc (Figure 3D). When we split this analysis in individual phenotypic groups, we found that the fraction of genes resulting in a mutant phenotype with significant expression varies from 89% (phenotypic class “nW”) to 69% (phenotypic class “WA”). This analysis is presented in Figure 3E and Supplementary Table S2. The phenotypes observed for genes that were classified as not expressed in the wing disc may be caused by misclassification of genes expressed at low levels and considered as not expressed based on RNA-Seq or microarray data. In addition, some cases of genes apparently not expressed in the wing disc but causing a mutant phenotype in knockdown condition could correspond to genes that are only expressed during the pupal stage. It is at this stage where the requirement for genes affecting wing shape and dorso-ventral adhesion could be maximal. Another fraction of not-expressed genes whose knockdown results in pupal lethality could be caused by the prominent expression of *nub-Gal4* in the larval nervous system. Finally, it is also expected that the phenotypes caused by off-target effects are within the group of genes not expressed but with a mutant phenotype. Some examples of *in situ* hybridization patterns and gene expression values are shown in Figure 3F.

### Phenotype comparison when using more than one UAS-RNAi strain to target the same gene

We are aware that some of the phenotypes we found could correspond to off-target effects arising through processed dsRNA that target unintended mRNAs by means of incomplete base pairing. The estimated rate of false positives in a background of Dicer2 overexpression is around 6% (Kulkarni *et al.* 2016). For a set of 281 genes, we used two or more independent RNAi lines (Supplementary Table S2). We observed that in 72% of the cases (202 genes), the resulting phenotypes were similar using different RNAi strains. In the remaining 28% of cases (79 genes), we found different results comparing two different RNAi lines directed against the same gene (Supplementary Table S2). In most of these cases (82%) one *nub-Gal4/UAS-RNAi* genotype resulted in wild-type flies whereas the other combination gave lethality or adult flies with a visible phenotype (Supplementary Table S2). We did not find cases in which two RNAi lines targeting the same gene resulted in opposite phenotypes (*e.g.*, large *vs* small wing size or extra *vs* loss of veins). These results suggest that a considerable fraction of discrepant results could be due to differential efficiency of independent *UAS-RNAi* lines targeting the same gene.

### Analysis of folded wings caused by overexpression of Tiptop in KK strains

**Figure 4:**
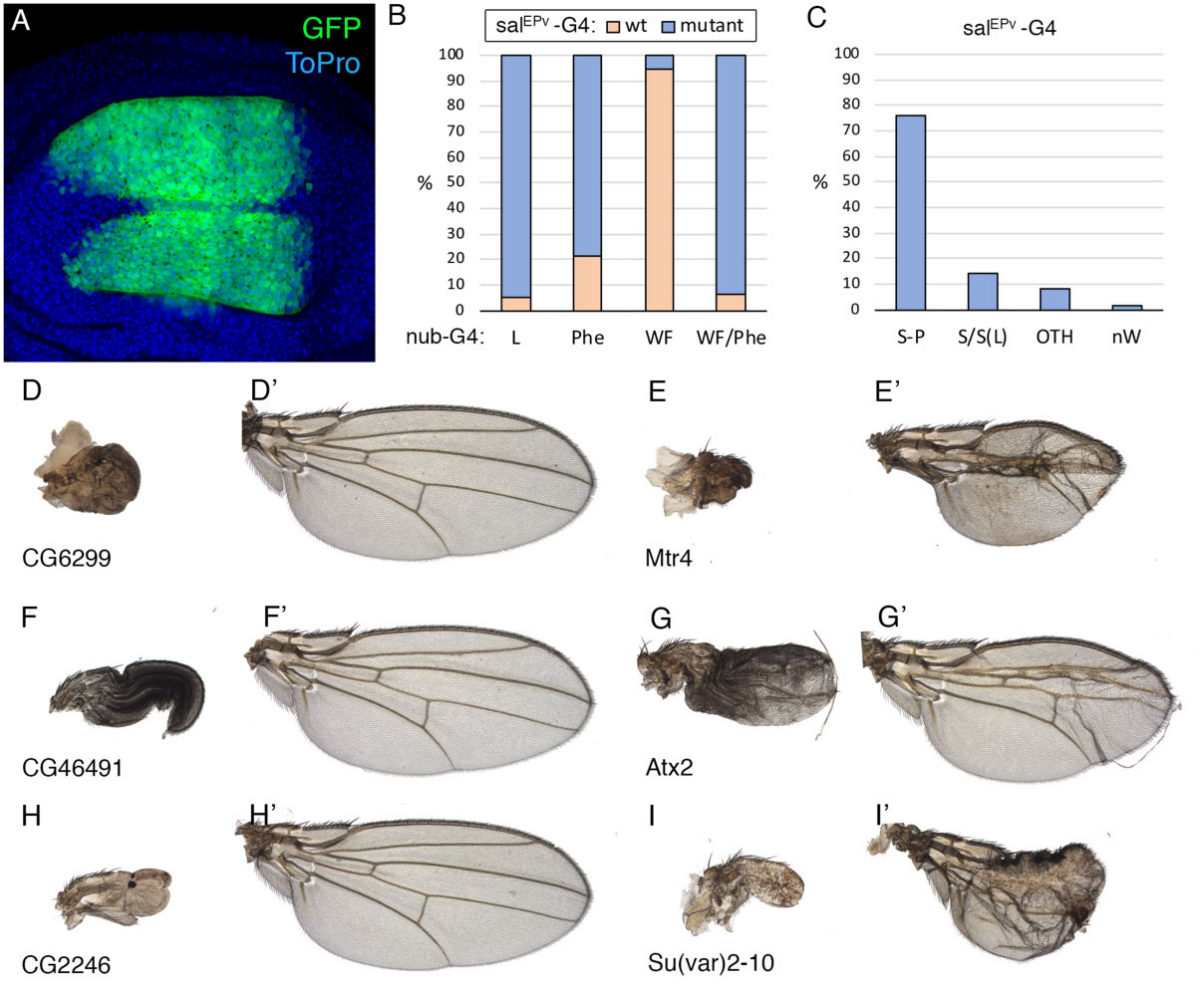
Analysis of *sal^EPv^-Gal4/UAS-RNAi* combinations. (A) Expression pattern of the *sal^EPv^-Gal4* driver (GFP, green) in third instar wing imaginal disc of *sal^EPv^-Gal4 UAS-GFP/+*. Nuclei are stained with ToPro (blue). (B) Phenotypic frequency of *UAS-Dicer2/+; sal^EPv^-Gal4/UAS-RNAi* combinations resulting in a mutant phenotype (blue section of each column) or wild-type wings (red section of each column) from UAS-RNAi lines that gave lethality (L), a visible phenotype (Phe), folded wings without additional phenotypes (WF), and folded wings with an additional phenotype (WF/Phe) in *UAS-Dicer2/+; nub-Gal4 UAS-RNAi/+* combinations. (C) Fraction of *sal^EPv^-Gal4 UAS-RNAi/+* mutant phenotypes observed with *UAS-RNAi* lines that were lethal in combination with *nub-Gal4*. OTH: other phenotypes (V+, V−, WA, WD, WM, WP). (D–I) Examples of wings showing the phenotype of combinations of the same *UAS-RNAi* line with *nub-Gal4* (left) and *sal^EPv^-Gal4* (right). (D–D’) *UAS-Dicer2/+; nub-Gal4/UAS-CG6299-i* (D) and *UAS-Dicer2/+; sal^EPv^-Gal4/UAS-CG6299-i* (D’). (E–-E’) *UAS-Dicer2/+; nub-Gal4/UAS-Mtr4-i* (E) and *UAS-Dicer2/+; sal^EPv^-Gal4/UAS-Mtr4-i* (E’). (F–F’) *UAS-Dicer2/+; nub-Gal4/UAS-CG46491-i* (F) and *UAS-Dicer2/+; sal^EPv^-Gal4/UAS-CG46491-i* (F’). (G–G’) *UAS-Dicer2/+; nub-Gal4/UAS-Atx2-i* (G) and *UAS-Dicer2/+; sal^EPv^-Gal4/UAS-Atx2-i* (G’). (H–H’) *UAS-Dicer2/+; nub-Gal4/UAS-CG2246-I* (H) and *UAS-Dicer2/+; sal^EPv^-Gal4/UAS-CG2246-i* (H’). (I–I’) *UAS-Dicer2/+; nub-Gal4/UAS-Su(var)2-10-i* (I) and *UAS-Dicer2/+; sal^EPv^-Gal4/UAS- Su(var)2-10-i* (I’).

It should be noticed that a significant proportion of the VDRC KK *UAS-RNAi* strains (∼25%) contain a P{UAS} insertion in the proximity of the gene *tiptop* (Green *et al.* 2014; Vissers *et al.* 2016). Recruitment of Gal4 to these *UAS* sequences causes overexpression of the transcription factor Tiptop, and this could lead to the formation of adults with folded wings (Green *et al.* 2014). We found a total of 1559 *UAS-RNAi* lines resulting in this “Wing Folded” phenotype (“WF”) in combination with *nub-Gal4* (Supplementary Table S1). In 22% of these cases (*n* = 352), we also observed a fraction of adult flies with unfolded wings displaying defects in the hinge and in the shape of the wing, consistent with being caused by *tiptop* overexpression (Supplementary Table S1). All these wings [“WF(s)/ds”] not showing any other additional phenotype were classified as wild-type wings. To better understand the phenotypes of *UAS-RNAi* lines causing a fully penetrant WF phenotype in combination with *nub-Gal4*, we crossed 138 of these *UAS-RNAi* lines with the driver *sal^EPv^-Gal4*, whose expression is restricted to the central region of the wing blade located between the veins L2 and L5 that does not include the wing hinge (Figure 4A). The wings of 131 out of these 138 *UAS-Dicer2/+; sal^EPv^-Gal4/UAS-RNAi* combinations were normal (95%), showing neither the WF nor any other phenotype (Supplementary Table S2; Figure 4, B and F–F’), indicating that the genes targeted by the RNAi are in most cases dispensable in the wing. Finally, we were able to identify 48 *nub-Gal4/UAS-RNAi* combinations where we could recognize size, trichome or cuticular differentiation phenotypes in wings with the typical “WF” appearance (Supplementary Table S1). In 15 out of 16 tested cases, these phenotypes affecting the wing independently of its folding were also recognized in *sal^EPv^-Gal4/UAS-RNAi* flies (Figure 4B). For all these reasons, we considered in all our quantifications as wild-type phenotypes all the cases of “WF” wings in which we could not identify any additional defect in wing morphology. We did not attempt to separate genetically the effects of the *tiptop* P{UAS} and UAS-RNAi insertions (see Vissers *et al.* 2016).

### Comparison of knockdown phenotypes using *sal^EPv^-Gal4 and nub-Gal4*

In aggregate, we analyzed a total of 722 *sal^EPv^-Gal4/UAS-RNAi* combinations. A subset of these *UAS-RNAi* lines (60%; *n* = 433) was chosen because they resulted in lethality or strong wing folded phenotypes in combination with *nub-Gal4*. The rest of these *UAS-RNAi* lines (40%; *n* = 289) were chosen at random. Approximately 82% of the genes included in this analysis (*n* = 722) were considered as expressed in the wing imaginal disc (*n* = 589; Supplementary Table S2). As expected, most RNAi lines resulting in normal flies in combination with *nub-Gal4* also gave normal-looking wings in combination with *sal^EPv^-Gal4* (96%; 190 out of 198 cases; Supplementary Table S2). *UAS-RNAi* lines resulting in lethality or absence of wings in combination with *nub-Gal4* (*n* = 289) affected in combination with *sal^EPv^-Gal4* wing size and pattern (73%; *n* = 211) or wing size (12%; *n* = 34), with 5% of combinations without any visible phenotype (*n* = 17; Figure 4C). Finally, 78% from 167 *UAS-RNAi* combinations giving a visible phenotype with *nub-Gal4* resulted also in a mutant phenotype in combination with *sal^EPv^-Gal4* (*n* = 131; Supplementary Table S2; Figure 4B). Examples of the phenotypes obtained in combinations of *nub-Gal4 and sal^EPv^-Gal4* with the same *UAS-RNAi* are shown in Figure 4, D–I. The examples of *Mtr4 helicase* (*Mtr4*; Figure 4, E–E’), *Ataxin-2* (*Atx2*; Figure 4, G–G’) and *Suppressor of variegation 2-10* [*Su(var)2-10*; Figure 4, I–I’] illustrate the cases more frequently found, in which phenotypes of loss of wing in combination with *nub-Gal4* correspond to phenotypes of defects in wing size and pattern in combination with *sal^EPv^-Gal4.* The example of *CG46491* (Figure 4, F–F’) illustrates the overwhelming majority of *UAS-RNAi/nub-Gal4* folded wings, which in the corresponding *UAS-RNAi/sal^EPv^-Gal4* combinations develop normal wings. Other less frequent cases are those of *UAS-RNAi* lines that in combination with *nub-Gal4* result in a strong phenotype and in combination with *sal^EPv^-Gal4* develop a normal wing (*CG6299*; Figure 4, D–D’ and *CG2246*; Figure 4, H–H’).

### The *Drosophila* genome: functional categories

**Figure 5:**
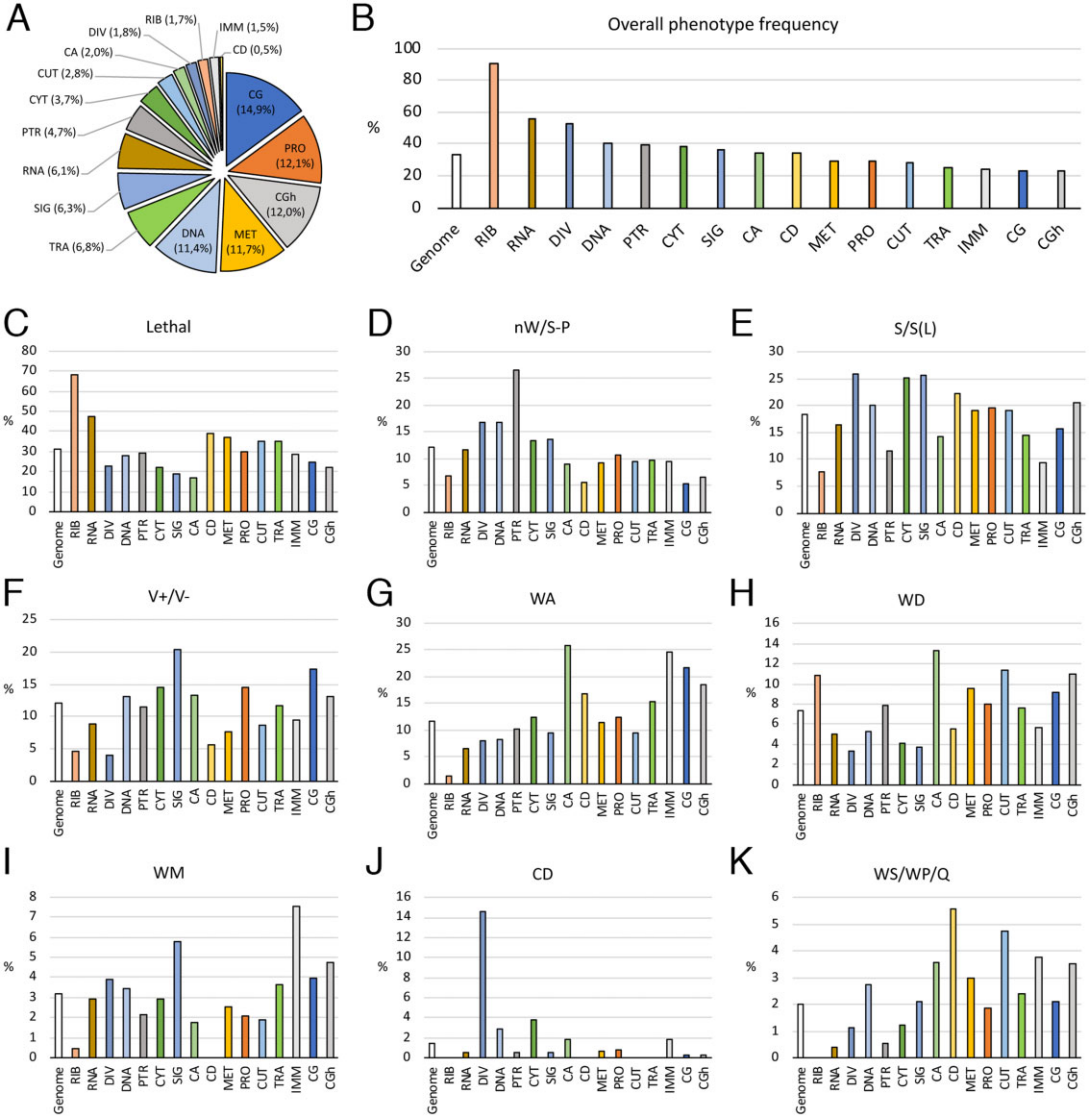
Classification of *Drosophila* genes into functional classes and examples of phenotypic frequencies within classes. (A) Percentage of protein-coding genes included in the molecular/functional classes “CG” (dark blue; 2084 genes) , “PRO” (Protein biology; orange; 1689 genes), “CGh” (dark gray; 1675 genes), “MET” (Metabolism; light orange; 1631 genes), “DNA” (DNA biology; light blue; 1598 genes), “TRA” (Transport; light green; 954 genes), “SIG” (Signaling; blue; 880 genes), “RNA” (RNA biology; brown; 851 genes), “PTR” (Protein transport; gray; 659 genes), “CYT” (Cytoskeleton; dark green; 513 genes), “CUT” (Cuticle; light blue; 387 genes), “CA” (Cellular adhesion; light green; 277 genes), “DIV” (Cell division; light gray; 245 genes), “RIB” (Ribosome; pink; 235 genes), “IMM” (Immune responses; gray; 216 genes), and “CD” (Cell death; yellow; 63 genes). (B) Percentage of genes in the screen causing a lethality or a visible phenotype in each molecular class. (C–K) Percentage of genes with a particular phenotype in each molecular class compared to the same values for the entire genome (Genome, white columns). Each column represents for each molecular class and the genome the frequency of lethality (Lethal; C), loss of wing and/or defects in wing size and pattern (nW/S/P; D), changes in wing size [S/S(L); E], defects in vein formation (V; F), failures in the adhesion between the dorsal and ventral wing surfaces (WA; G), defects in wing cuticular differentiation (WD; H), partial loss of wing margin structures (WM; I), Trichome differentiation (CD; J) and other less frequently observed phenotypes (WS/WP/Q).

Wing phenotypes reveal functional requirements, either in basic cellular functions impinging on cell viability or in more wing-specific functions related to the growth and patterning of the wing imaginal disc. In order to relate each phenotype with the predicted function of the corresponding gene, we wanted to define for each *Drosophila* gene a single term summarizing its molecular function. To do this, we first used Flybase and Flymine to compile all GO and IP terms available for each gene. Subsequently, we summarized this information to classify each gene into 1 of 14 functional categories that we thought encompass the most relevant aspect of each gene/protein function. These categories are “Cell adhesion” (CA), “Cell death” (CD), “Cuticular differentiation” (CUT), “Cytoskeleton organization” (CYT), “Cell division” (DIV), “Ribosome function” (RIB), “Cell signaling” (SIG), “Transport across cell membranes” (TRA), “Protein trafficking” (PTR), “Cellular metabolism” (MET), “Immune Responses” (IMM), “DNA Biology” (DNA), “RNA Biology” (RNA), and “Protein modifications” (PRO) (see Table 1 for abbreviations). To these 14 groups, we added two groups including those genes for which there is no information based in sequence or functional approaches (CG) and all genes for which there is at least one IP domain defined (CGh). The number and fraction of genes included in each molecular class are presented in Table 2 and Figure 5A, respectively. Using this classification, we analyzed phenotypic frequencies within each molecular/functional class. We found that some classes are much more likely to contain genes whose knockdown results in lethality or a mutant phenotype in the wing. The molecular classes “RIB” (90%; 178/190), “RNA” (56%; 391/702), and “DIV” (52%; 114/218) have a frequency of genes with an altered phenotype way above the 33% average observed for the total of 10,920 genes tested (*n* = 3653; Figure 5B). Conversely, the molecular classes “TRA” (25%; 199/797), IMM (24%; 41/169), “CG” (23%; 276/1181), and CGh (23%; 299/1294) have lower percentages of genes with knockdown phenotypes (Figure 5B).

In general, the most prevalent visible phenotypes, such as those affecting the size of the wing, its growth and pattern, the adhesion between the dorsal and ventral wing surfaces, the differentiation of cuticle and the formation of the wing margin, are observed in all molecular classes (Figure 5, C–K). We also found specific enrichment for several phenotypes in specific molecular classes. For example, lethality was particularly enriched in the “RIB” class (Figure 5C), defects in wing growth and patterning in the “PTR” class (Figure 5D), wings of reduced size and abnormal cell size were more frequent in the “DIV,” “CYT,” and “SIG” classes (Figure 5E), defects in wing vein and wing margin formation (V+/V- and WM) were prevalent in the “SIG” class (Figure 5 F and I), wings with dorso-ventral adhesion failures were more frequently found in the “CA” class (Figure 5G) and defects in trichome differentiation (“CD”) were particularly prominent in the “DIV” class (Figure 5J). A more detailed phenotypic analysis of the different molecular classes is presented in the accompanying manuscript (López-Varea *et al.* 2021).

**Figure 6:**
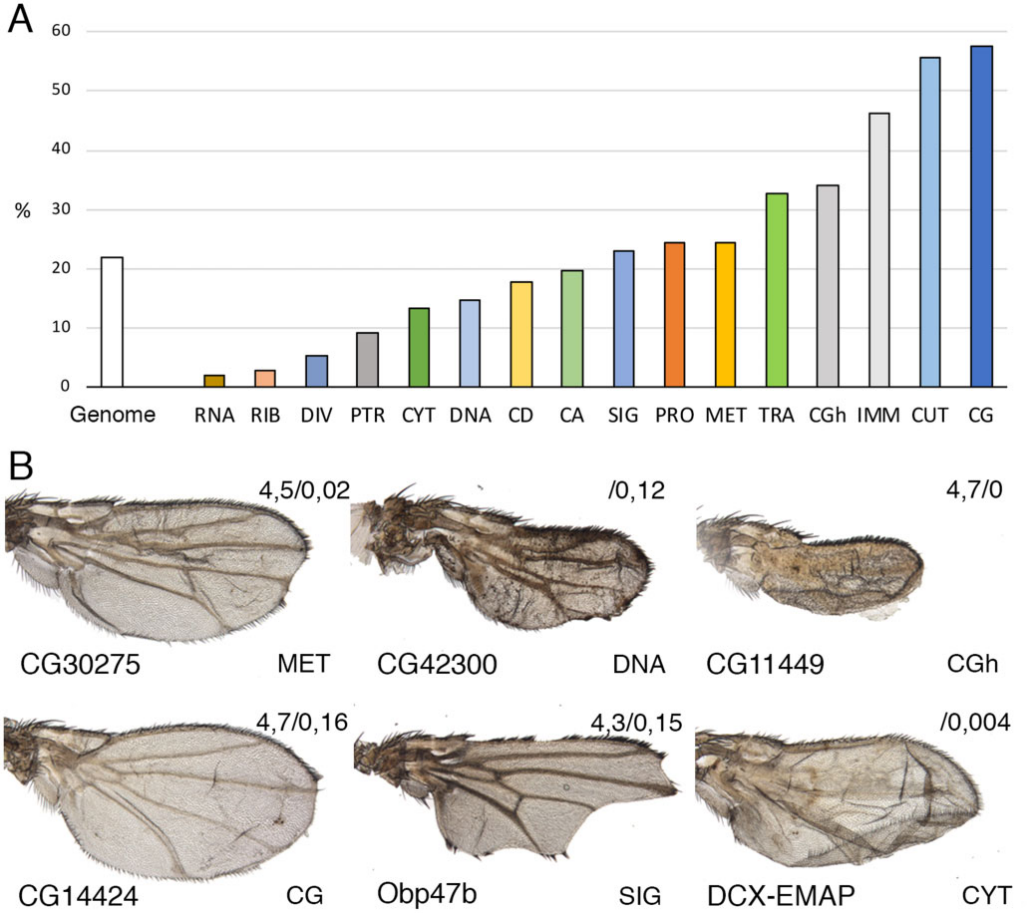
Phenotypic frequencies for genes not expressed in the wing disc. (A) Percentage of lethality or visible phenotypes in *UAS-Dicer2/+; nub-Gal4/UAS-RNAi* flies for genes that were considered as not being expressed in the wing disc. The white column is for the total number of genes not expressed and the colored columns from left to right for genes grouped in the molecular classes RNA, RIB, DIV, PTR, CYT, DNA, CD, CA, SIG, PRO, MET, TRA, CGh, IMM, CUT, and CG. (B) Example of wings with a mutant phenotype from knockdown of genes (name below each wing) not expected to be expressed in the wing disc. The values of expression intensity detected in Affimetrix and RNA-Seq experiments (Affimetrix/RNA-Seq) are indicated in the upper right corner of each picture. The molecular class of each gene is indicated in the lower-right corner of each wing.

The fraction of genes apparently not expressed in the wing disc but showing a phenotype in the wing is 22% in the genome (800/3653). We notice that this fraction varies considerably when comparing different molecular classes (Figure 6A). Thus, this value is minimal for genes of the classes RIB, DIV, and PTR (3.1%, 5%, and 7.5%, respectively; Figure 6A), and maximal for the CGh, IMM, CUT, and CG classes (34%, 46%, 54%, and 57%, respectively; Figure 6A). The phenotypes observed for genes apparently not expressed in the wing disc belong to the same classes identified for expressed genes (see some examples in Figure 6B).

### Examples of individual genes

**Figure 7:**
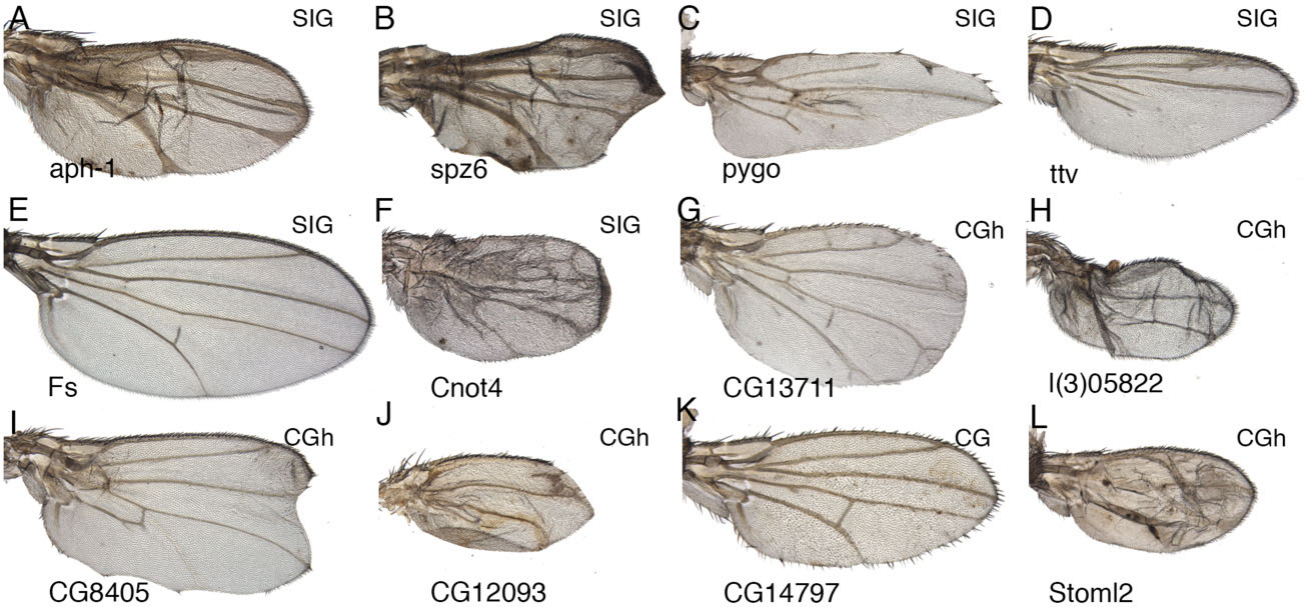
Examples of wing phenotypes for the SIG, CGh, and CG functional classes. (A) *UAS-Dicer2/+; nub-Gal4/UAS-aph1-RNAi.* (B) *UAS-Dicer2/+; nub-Gal4/UAS-spz6-RNAi.* (C) *UAS-Dicer2/+; nub-Gal4/UAS-pygo-RNAi.* (D) *UAS-Dicer2/+; nub-Gal4/UAS-ttv-RNAi*. (E) *UAS-Dicer2/+; nub-Gal4/UAS-Fs-RNAi*. (F) *UAS-Dicer2/+; nub-Gal4/UAS-Cnot4-RNAi*. (G) *UAS-Dicer2/+; nub-Gal4/UAS-CG13711-RNAi.* (H) *UAS-Dicer2/+; nub-Gal4/UAS-l(3)05822-RNAi*. (I) *UAS-Dicer2/+; nub-Gal4/UAS-CG8405-RNAi*. (J) *UAS-Dicer2/+; nub-Gal4/UAS-CG12093-RNAi*. (K) *UAS-Dicer2/+; nub-Gal4/UAS-CG14797-RNAi*. (L) *UAS-Dicer2/+; nub-Gal4/UAS-Stoml2-RNAi*. The functional class of each gene is indicated in the upper right corner of each picture.

Phenotypic information is a key entry point in the analysis of gene function, as it informs about the potential function of a gene in a particular tissue context. We show in Figure 7, some examples of the phenotypes we observed for genes that we annotated as being expressed in the wing disc. In some cases, the observed phenotypes are reminiscent of those caused by alterations in the activity of the signaling pathways regulating wing growth, wing margin formation, and vein patterning and differentiation (Molnar *et al.* 2011). For example, loss of Notch activity in the wing disc results in vein thickening and loss of wing margin formation similar to loss of *anterior pharynx defective 1* (*aph-1*; Figure 7A) and *spatzle 6* (*spz6*; Figure 7B). Consistently, *aph-1* encodes a scaffolding subunit of the gamma-secretase complex participating in the processing of the Notch receptor (Shih and Wang 2007). In contrast, *spz6* encodes a secreted protein of the Spatzles family that is known to activate Toll signaling and so far has not being linked to Notch signaling (Lewis *et al.* 2013). Phenotypes presenting loss of veins are reminiscent of loss of Dpp/BMP (Decapentaplegic/bone morphogenetic protein) or EGFR (epidermal growth factor receptor) signaling (de Celis 2003). *pygopus* (*pygo*) encodes a nuclear component of the Wg (Wingless)/Wnt-βcatenin signaling pathway (Belenkaya *et al.* 2002) and its phenotype in the wing margin is compatible with reduced Wg signaling (Figure 7C). Knockdown of *pygo* also causes loss of veins, a phenotype that is not related to Wg signaling (Figure 7C). *tout-velu* (*ttv*) encodes a Glucuronosyltransferase involved in the synthesis of heparan sulfate proteoglycans, which are required for Wingless, Hedgehog, and Decapentaplegic signaling (Bornemann *et al.* 2004). In our hands, knockdown of *ttv* only appears to compromise Decapentaplegic signaling, because its knockdown (Figure 7D) results in a phenotype characteristic of *thick veins* loss-of-function alleles (de Celis 1997). The case of *Follistatin* (*Fs*), encoding a secreted protein that inhibits Activin ligands (Pentek *et al.* 2009), is also intriguing. On the one hand, knockdown of *Fs* results in larger than normal wings, compatible with increased Activin signaling (Figure 7E). In addition, these wings also show loss of crossveins, a phenotypic trait characteristic of reduced Decapentaplegic signaling (Figure 7E). Another interesting example of a gene belonging to the signaling class is *CCR4-NOT transcription complex subunit 4* (*Cnot4*), which encodes a positive regulator of the Jak/Stat signaling pathway (Grönholm *et al.* 2012). Knockdown of *Cnot*4 causes a phenotype of ectopic vein formation (Figure 7F), which suggests ectopic EGFR signaling. Knockdown of genes belonging to the CG and CGh classes also could result in informative phenotypes (Figure 7, G–L), which would help to identify their functions. For example, *CG7129* was identified as a modifier of receptor tyrosine kinase signaling (Zhu *et al.* 2005), and its phenotype in the wing includes the formation of ectopic veins (Figure 7H). Similarly, *CG8405* was also identified in a gain-of-function screen searching for suppressors of *Beadex* (Bejarano *et al.* 2008), and its loss-of-function phenotype in the wing includes defects in the formation of the wing margin (Figure 7I). In other instances, for example, *CG12093* (Figure 7J), *CG14797* (Figure 7K), and *Stomatin-like 2* (*Stoml2*; Figure 7L), no previous information is available. The loss-of-function phenotypes we observe are indicative of a requirement of these genes for wing growth and vein patterning. A more detailed phenotypic description and gene functional classification are presented in the accompanying manuscript (López-Varea *et al.* 2021).

### Correlation with other genome-wide RNAi screens

**Figure 8:**
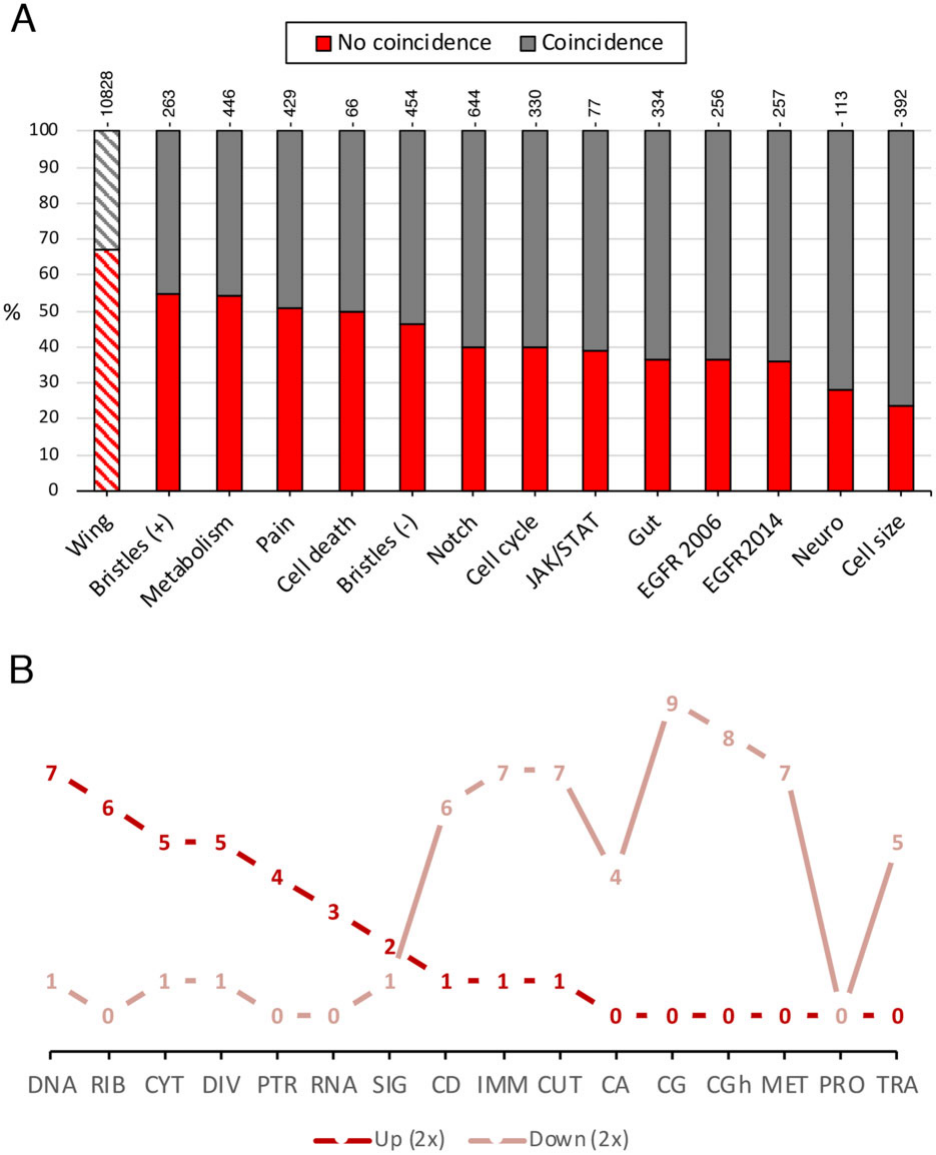
Correlation of the wing screen with other RNAi genetic screens. (A) The left column represents the percentage of genes with lethality or visible phenotype in *UAS-Dicer2/+; nub-Gal4/UAS-RNAi* (striped gray section) or without any phenotype (striped red section). The following columns represent the percentage of genes identified in each screen that also give a phenotype in the wing screen (gray section; coincidence). The percentage of genes identified in each screen that do not give a phenotype in the wing screen is represented in the red section of each column (No coincidence). (B) Number of times that a particular molecular class (DNA, RIB, CYT, DIV, PTR, RNA, SIG, CD, IMM, CUT, CA, CG, CGh, MET, PRO, and TRA) appear over-represented (2x) or under-represented (2x) in 11 independent screens with respect to the fraction of genes included in each molecular class in the genome. Dark red lines indicate over-representation and light red lines under-representation.

The development of the wing requires the contribution of signaling pathways and general cellular functions that are common to many other developmental systems. For this reason, we compared our results with those of other genome-wide RNAi screens carried out either in cell cultures or addressing particular physiological processes. The screens we considered were aimed at identifying genes regulating the cell cycle (Björklund *et al.* 2006), cell death (Chew *et al.* 2009), EGFR signaling (Friedman and Perrimon 2006; Ashton-Beaucage *et al.* 2014), Notch signaling and bristle formation (Mummery-Widmer *et al.* 2009), Notch signaling (Saj *et al.* 2010), JAK/STAT signaling (Baeg *et al.* 2005), Metabolism (Reed *et al.* 2014), heat nociception (Neely *et al.* 2010), cytoskeletal organization (Bai *et al.* 2011), neuromuscular junction (Valakh *et al.* 2012), and intestinal stem cell regulation (Zeng *et al.* 2015). We first aimed to find whether the genes that give a phenotype in these 12 independent published screens (Supplementary Table S3) were also identified in our screen as causing lethality or changes in wing morphology, size, or pattern. The percentage of genes whose knockdown causes lethality or a phenotype in the wing is 33% (see Figure 8A, left column). In case of each screen identifying an independent set of genes giving a phenotype, we expect for each set a similar ratio of genes showing a phenotype or lethality in our screen. In contrast, we found much higher ratios in pair-wise comparisons between our screen and these screens, varying from 45% to 76% of genes identified in our screen that were also identified in these independent screens (Figure 8A). In addition, we explored whether the molecular classes identified in all these independent screens were similarly enriched. We found this to be the case, with some molecular categories mostly under-represented in all screens (CA, CD, CG, CGh, CUT, IMM, MET, and TRA; Figure 8B) and others mostly over-represented (CYT, DIV, DNA, PTR, RIB, RNA, and SIG) in several independent screens (Figure 8B).

In summary, we screened a collection of *UAS-RNAi* lines targeting 10,920 *Drosophila* protein-coding genes for phenotypes in the adult wing. We classified the resulting phenotypes in the wing into morphological classes affecting the size, pattern, or differentiation of the wing, and correlated each mutant phenotype in the wing with the expression levels of the corresponding gene in the wing disc. Using existing GO and IP annotations, we present a grouping of *Drosophila* genes into 16 functional groups encompassing the more relevant aspect of each gene. A more in-depth analysis of these functional classes is presented in the accompanying manuscript (López-Varea *et al.* 2021).

## Data availability

The data underlying this article are available in the article and in its online supplementary material. All wing pictures we have were submitted to the Figshare repository: https://doi.org/10.6084/m9.figshare.16624645.v1; https://doi.org/10.6084/m9.figshare.16624630.v1; https://doi.org/10.6084/m9.figshare.16624603.v1; and https://doi.org/10.6084/m9.figshare.16624591.v1

## Supplementary Material

jkab351_Supplementary_Data
