# Supplementary material for: Genome-wide phenotypic RNAi screen in the Drosophila wing: global parameters
Source: G3 (Bethesda). 2021 Oct 2;11(12):jkab351. doi: 10.1093/g3journal/jkab351 (PMC8962446; doi:10.1093/g3journal/jkab351)
Supplement: jkab351_Supplementary_Data [file jkab351_supplementary_data.zip › GENETICS-G3-2021-402806-s01.docx]

**Supplementary** **Table 1**

Results of the RNAi screen.

RNAi strain (TransformantID), Flybase identifiers (FlybaseID), Gene names (CGNR and Gene symbol), Phenotypic description (Phenotype) using the abbreviations described in Table 1. MN column: Molecular classes as defined in the text and Table 1. Expression Affi: Average expression level in Affimetrix microarray, Expression RNAs: tpm in RNAseq data. Expression: Positive (Y) and negative (N) expression in the wing disc.

**Supplementary** **Table 2**

**two RNAi**: Results for genes targeted by two or more UAS-RNAi lines.

**nub vs sal**: Results for UAS-RNAi lines crossed with *nub-Gal4* and *sal^EPv^-Gal4*. Data for each driver separated by // in each table cell.

**Expression vs *in situ***: Expression data (Y and N; expression positive and negative, respectively) and *in situ* data (P: spatial pattern, G: generalized expression and N: expression don’t detected) for a collection of 562 genes.

**Supplementary** **Table 3**

Genes identified in 12 independent screens compared to the wing screen

**Supplementary** **Table 4**

List of abbreviations in MS Exel format
